# Performance Analysis of Double-Layer Microchannel Heat Sinks under Non-Uniform Heating Conditions with Random Hotspots

**DOI:** 10.3390/mi8020054

**Published:** 2017-02-14

**Authors:** Danish Ansari, Kwang-Yong Kim

**Affiliations:** Department of Mechanical Engineering, Inha University 100, Inha-Ro, Nam-Gu, Incheon 22212, Korea; danishansari@live.in

**Keywords:** electronic cooling, double-layer microchannel heat sink, random hotspots, transverse-flow, Latin hypercube sampling

## Abstract

Performance analysis of double-layer microchannel heat sinks was performed under non-uniform heating conditions having randomly distributed hotspots. Two parallel-channel (parallel-flow and counter-flow) and one cross-channel (transverse-flow) designs of double-layer heat sink were evaluated with three sets of heating schemes. Each set of heating scheme consisted of eleven randomly distributed hotspots generated by Latin hypercube sampling. The heat flux, area, and location of the hotspots were selected as the design parameters. Conjugate heat transfer analysis of the heat sinks was performed by solving three-dimensional Navier–Stokes and energy equations. Water with temperature-dependent properties was selected as the coolant. The thermal resistance, pressure drop, maximum temperature rise, and temperature variation among hotspots were evaluated for all the heat sinks. The transverse-flow microchannel heat sink exhibited the lowest thermal resistance, temperature rise and temperature variation among the hotspots throughout the specified range of flow rate. The lowest pressure drop was exhibited by the counter-flow heat sink.

## 1. Introduction

A microprocessor is composed of various blocks producing different amounts of heat, and the logic-block being the highest contributor creates a localized high heat flux region known as “hotspot” [1]. Design complexity and dynamic power dissipation in a single core processor caused the design architecture to shift towards multicore [2]. In a multicore processor, most of the heat is concentrated at the cores creating multiple hotspots. High temperatures at the hotspots create large temperature gradients which induce thermal stresses creating circuit imbalances that could affect the efficiency and service life of a microprocessor [3]. With the advancement in manufacturing techniques [4], a microprocessor with 5.4 billion transistors was recently developed at IBM [5]. The thermal design powers (TDPs) of central processing units (CPUs) and graphics processing units (GPUs) have reached 150 W and 250 W, respectively [6]. Air-cooling techniques have reached their limits [7], and thus there is an eminent need to explore more effective cooling solutions that can keep up with the heat dissipation requirement of the advanced microprocessors.

Tuckerman and Pease [8] showed promising results by using a microchannel heat sink with water as a coolant. Following their work, numerous studies have been presented to explore and enhance the thermo-hydraulic performance of microchannel heat sinks. Liu and Garimella [9] performed numerical and experimental study on fluid flow in microchannels with wide ranges of hydraulic diameters and Reynolds numbers. They concluded that the conventional Navier–Stokes theory can be employed using commercial software packages to understand the flow in microchannels. Several active [10,11] and passive [11,12,13,14] heat transfer augmentation techniques have been explored to enhance the heat transfer capacity. Passive techniques (as considered in the present work) are the preferred choice over active techniques as they have simple design and also do not require external power therefore eliminating additional complexity and costs [11]. 

Double-layer microchannel heat sink gained attention for its higher heat transfer capability over single-layer microchannel heat sink [15,16]. A better temperature uniformity is offered by the double-layer counter-flow arrangement as compared to the double-layer parallel-flow arrangement [17]. Performance enhancements of double-layer microchannel heat sinks were reported by using converging channels [18,19] and passive microstructures [12,13,14,20], and by optimizing channel shapes [21,22,23]. Along with double-layer parallel-flow and counter-flow heat sinks, Ansari and Kim [24] analyzed the performance of a double-layer transverse-flow heat sink design with various flow configurations. They reported that the transverse-flow heat sink showed better temperature uniformity and lower thermal resistance as compared to double-layer parallel-flow and counter-flow heat sinks. Use of temperature-dependent fluid properties for numerical analysis is recommended by many researchers unless the temperature difference is very small as the constant fluid property method is crude approximation of the real phenomenon [25,26].

In a single core processor, the maximum clock frequency has saturated around 4 GHz due to increased design complexity [2]. Since 2004, the processor architecture has shifted towards multicore to keep up with the increasing requirement for computational capability [2,27]. The high heat flux concentrated at the cores as compared to the remaining chip area [28,29,30,31], creates large temperature gradient across the chip which leads to circuit imbalances and eventually degradation in the microchip performance [32]. Using spatially-resolved imaging of microprocessor power (SIMP) technique, Hamann et al. [33] analyzed the power distribution of a fully working dual core microprocessor. They presented that the flux intensity as well as the actual position of a hotspot in a dual core microprocessor changes quite drastically depending upon the level of utilization.

Hotspots in a microchip are currently being identified as a prevalent challenge in the design of an efficient heat sink. If heat sinks are designed considering the maximum heat flux at the hotspots, it would lead to over-cooling of the remaining background area of the microchip, while designing it considering average heat flux generated by microchip would lead to under-cooling of high heat flux regions. Abdoli et al. [34] numerically investigated performance of a micro pin-fin array heat sink with a single hotspot at the center and presented the effects of the pin-fin shape and the height on the thermo-hydraulic performance using six different pin-fin shapes. Effect of manifold design on the performance of a microchannel heat sink with non-uniform heating was presented by Liu et al. [35]. Various cooling techniques have been proposed for hotspot management using minichannel heat sink and thermoelectric cooler [36], hierarchical manifold microchannel heat sink [37], and micro heat sink with non-uniform pin-fin distribution [38]. Feng et al. [39] performed an analysis of a pin-fin heat sink under non-uniform impingement heating, and presented an analogy model for the fast calculation of thermal resistance with reasonable accuracy as compared to numerical calculation.

With maximum clock frequency of a single core processor at its limits [2], almost all future CPUs and GPUs would have multiple cores, creating multiple high heat flux zones. In addition, the intensity and the location of high heat flux varies with the processors utilization [33]. Thermal analysis of heat sinks considering random non-uniform heating schemes, would closely imitate the actual heating conditions of future multicore processors, and the results would be more reliable and practical. In the present study, performance of a previously proposed flow configuration of double-layer transverse-flow microchannel heat sink [24], was evaluated under random non-uniform heating condition and compared with that of double-layer parallel-flow and counter-flow heat sinks. Three different heating schemes with eleven random hotspots in each scheme were selected as heating conditions. Heat flux, area, and location were selected as the hotspots parameters. Latin hypercube sampling (LHS) [40,41] method was used to generate the hotspots in each set. Full heat sink was selected as the computational domain and a three-dimensional (3-D) conjugate heat transfer analysis was performed using Navier–Stokes and energy equations with temperature-dependent fluid properties. The performances of the heat sinks were evaluated in terms of maximum temperature rise at the hotspots, temperature variation among the hotspots, pressure drop and the total thermal resistance.

## 2. Heat Sink Models and Heating Conditions

**Figure 1:**
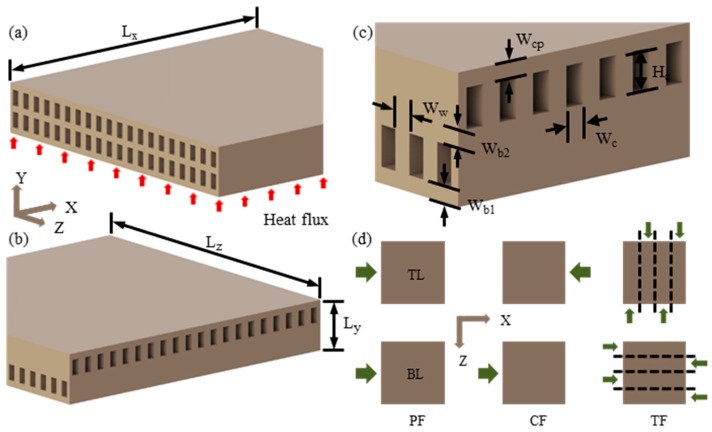
Schematic diagrams: (**a**) parallel-channel design (PF and CF); (**b**) cross-channel design (TF); (**c**) geometric parameters; and (**d**) flow conditions for top layer (TL) and bottom layer (BL).

The schematic configurations of two double-layer microchannel heat sinks (parallel-channel and cross-channel) analyzed under random non-uniform heating conditions are shown in Figure 1a,b. Each heat sink model was composed of total forty microchannels with twenty microchannels in each layer. In case of the parallel-channel design (Figure 1a), the channels in both the top and the bottom layers are aligned parallel to each other, whereas, in case of the cross-channel design (Figure 1b), the channels in the top layer are transversal (at right angles) to those in the bottom layer. To evaluate the performances of these two heat sink designs under non-uniform heating conditions, three flow configurations were selected. The two flow configurations from the parallel-channel design (PF and CF) and one flow configuration from the cross-channel design (TF) are shown in Figure 1d. In the PF configuration, the flow directions in both the bottom and the top layers are same. In the CF configuration, the flow direction in the top layer is opposite to that in the bottom layer. In the TF configuration, these two flow directions are transversal (at right angles); moreover, each layer is subdivided into quarters with opposite flow directions in the adjacent quarters. The TF flow configuration considered here showed the best performance among all the analyzed flow configurations of the parallel-channel and cross-channel designs in the previous study presented by the authors [24].

Both the heat sinks were designed with exactly same values of the geometric parameters as listed in Table 1. The analyzed heat sink designs can be manufactured using the process presented by Wei et al. [17]. Improper selection of inlet manifold could lead to non-uniformity in flow distribution resulting in high temperature rise [35] and increased pressure drop at high flow rates [35,42]. To uniformly distribute the coolant in microchannels, a multi-layer manifold design can be employed as presented by Wei et al. [17]. In this work, however, the inlet manifold design was not included in the computational domain due to the limitation of computational resources. Thus, to minimize the computational cost, the present research was concentrated to understand the effect of randomly distributed hotspots on the performance of the double-layer microchannel heat sinks.

**Figure 2:**
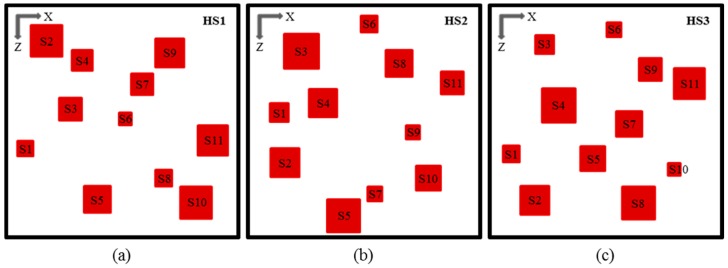
Hotspot layout schemes: (**a**) HS1; (**b**) HS2; and (**c**) HS3.

Three hotspot schemes (HS1, HS2, and HS3) were considered with eleven hotspots in each scheme, where the hotspots were generated randomly by using a design-of-experiment technique, LHS [41]. The heat flux (*q*_hs_), area (*A*_hs_), and location (*x* and *z*) of the hotspots were selected as the hotspot parameters. Table 2 shows the hotspot design parameters with their ranges. The hotspot design schemes, HS1, HS2, and HS3, are shown in Figure 2, and their hotspots are listed in Table 3, Table 4 and Table 5, respectively. In experimental case, the temperature measurements at the hotspots can be performed by utilizing infrared (IR) imaging technique using an IR-transparent heat sink as presented by Hamann et al. [33].

## 3. Numerical Analysis

A general purpose commercial CFD code, ANSYS CFX 15.0 [43] was used to perform three-dimensional conjugate heat transfer analyses in microchannel heat sinks. The code uses an implicitly coupled algebraic multigrid solver [44], which solves the governing equations for mass, momentum, and energy conservation. In the multigrid technique, the solver performs initial iterations on a fine mesh, then, on a coarser mesh which is created virtually, and finally the results are transferred back to the finer mesh to obtain accurate results. The coupled solver reduces the convergence time by solving an entire set of the equations simultaneously. The calculation of each equation was considered to have converged when the root-mean-square residual was less than 10^−6^.

Several assumptions were made to simplify the numerical calculation. Flow inside the microchannels was considered to be steady, incompressible, and laminar. The gravitational effect and radiation heat transfer were neglected. The steady-state governing equations for conjugate heat transfer in fluid and solid regions are describe as follows:

Continuity equation:

(1) $$\nabla \cdot \left(\rho_{f}\bar{V}\right)=0$$

Momentum equations:

(2) $$\bar{V}\cdot \nabla \left(\rho_{f}\bar{V}\right)=-\nabla p+\nabla \cdot \left(\mu_{f}\nabla \bar{V}\right)$$

Energy equation (liquid domain):

(3) $$\bar{V}\cdot \nabla \left(\rho_{f}C_{p}T_{f}\right)=\nabla \cdot \left(k_{f}\nabla T_{f}\right)$$

Energy equation (solid domain):

(4) $$\nabla \cdot (k_{s}\nabla T_{s})=0$$

To evaluate the performance of the heat sinks, total thermal resistance, maximum temperature rise at hotspots, temperature variation among hotspots, and total pressure drop, were calculated. The total thermal resistance of heat sinks under uniform as well as non-uniform heating conditions was defined as follows [8,39]:

(5) $$R_{\mathrm{th},\mathrm{tot}}=\frac{T_{s,\max}-\;T_{f,\mathrm{in}}}{Q_{\mathrm{tot}}}$$

where *T*_s,max_ is the maximum temperature at the bottom surface (solid) of heat sink, *T*_f,in_ is the inlet fluid temperature, and *Q*_tot_ is the total heat applied at the bottom surface (solid) of the heat sink.

The *Q*_tot_ for heat sink without hotspots was defined as:

(6) $$Q_{\mathrm{tot},\mathrm{WHS}}=q_{b}A_{b}$$

where *q*_b_ and *A*_b_ are the heat flux applied at the bottom surface and the total area of the bottom surface of the heat sink, respectively. For heat sink with hotspots, it was defined as:

(7) $$Q_{\mathrm{tot},\mathrm{HS}}=q_{\mathrm{bg}}A_{\mathrm{bg}}+\sum \left(q_{\mathrm{hs}}A_{\mathrm{hs}}\right)$$

where *q*_bg_ is the heat flux applied at the background area of the heat sink (area excluding the hotspots) and *A*_bg_ is the background area of the heat sink. *q*_hs_ and *A*_hs_ are the heat flux applied at each hotspot and the area of each hotspot, respectively. $\sum \left(q_{\mathrm{hs}}A_{\mathrm{hs}}\right)$ is the total heat applied at all (eleven) the hotspots. 

The total pumping power required to pump the coolant through the channels was calculated as:

(8) $$P_{\mathrm{tot}}=n_{\mathrm{ch}}u_{\mathrm{avg}}A_{c}\Delta p_{\mathrm{ch}}$$

where $n_{\mathrm{ch}}$ is the total number of channels in both layers, $u_{\mathrm{avg}}$ is the average inlet velocity of fluid, $A_{c}$ is the channels cross-sectional area, and $\Delta p_{\mathrm{ch}}$ is the pressure drop within a single channel.

The Reynolds number was defined as:

(9) $$Re=\frac{\rho_{f}u_{\mathrm{avg}}D_{h}}{\mu_{f}}$$

where ρ_f_ is the fluid (water) density, *D*_h_ is the channel hydraulic diameter, and μ_f_ is the dynamic viscosity of the fluid. 

In the case of cross-channel design (TF configuration) (Figure 1b), selection of a periodic or symmetric computational domain is not possible, while it is possible for the parallel-channel design (PF and CF configurations) (Figure 1a). However, to maintain the consistency in result presentation, the complete heat sinks were selected as the computational domain for all the flow configurations (PF, CF, and TF). As boundary conditions, a uniform mass flow rate was assigned at all the channel inlets (thus, the flow rates in the bottom and top layers are same), and atmospheric pressure condition was assigned at the outlets. A uniform heat flux (100 W/cm^2^) was applied at the bottom surface of heat sinks without hotspots (*q*_b_) and the same was used as the background heat flux (area excluding hotspots) of heat sinks with hotspots (*q*_bg_).

Water represents superior thermophysical properties [16], and thus it was selected as the coolant. The inlet temperature of water $\left(T_{f,\mathrm{in}}\right)$ and the heat sink (silicon) was set at 300 K. Silicon being the most widely used semiconductor material was selected as the heat sink material. The thermophysical properties of silicon, i.e., the density (ρ_s_), specific heat (*C*_p,s_), and the thermal conductivity (*k*_s_), were assigned as 2330 kg/m^3^, 712 J/kg·K, and 148 W/m·K, respectively [45].

The thermophysical properties of water were allowed to vary with temperature. Polynomial correlations, between the thermophysical properties of water and temperature in the property table [45], were developed and verified by using MATLAB [40] in this work. The correlations for density (ρ_w_), specific heat (*C*_p,w_), thermal conductivity(*k*_w_), and dynamic viscosity (μ_w_) are described as follows:

(10) $$\rho_{w}\left(T\right)=999.9+9.561\times 10^{-2}T-1.013\times 10^{-2}T^{2}+8.459\times 10^{-5}T^{3}-3.496\;10^{-7}T^{4}$$

(11) $$C_{p,w}\left(T\right)=4217-3.452T+1.155\times 10^{-1}T^{2}-1.862\times 10^{-3}T^{3}+1.538\times 10^{-5}T^{4}-4.850\times 10^{-8}T^{5}$$

(12) $$k_{w}\left(T\right)=5.698\times 10^{-1}+1.772\times 10^{-3}T-4.870\times 10^{-6}T^{2}-2.915\times 10^{-8}T^{3}+1.094\times 10^{-10}T^{4}$$

(13) $$\mu_{w}\left(T\right)=1.750\times 10^{-3}-5.558\times 10^{-5}T+1.172\times 10^{-6}T^{2}-1.579\times 10^{-8}T^{3}+1.169\times 10^{-10}T^{4}-3.535\;\times 10^{-13}T^{5}$$

where *T* is the temperature in degrees Celsius (°C).

**Figure 3:**
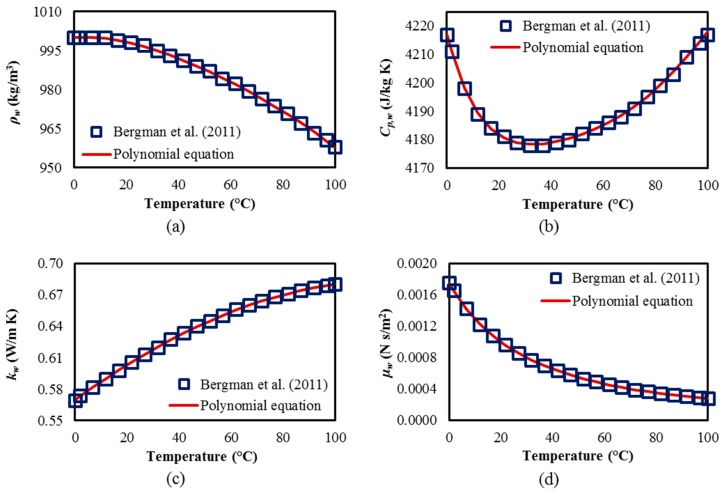
Verification of polynomial correlations between thermophysical properties of water and temperature with the property data from Bergman et al. [45]: (**a**) density ($\rho_{w}$); (**b**) specific heat ($C_{p,w}$); (**c**) thermal conductivity ($k_{w}$); and (**d**) dynamic viscosity ($\mu_{w}$).

The prediction accuracies of the above correlations were verified by comparing the predicted values obtained by the correlations with the values obtained from the property table [45], as shown in Figure 3. Excellent agreements between the predicted values from the correlations and the original data [45] can be observed in Figure 3. The maximum percentage residuals for the density, specific heat, thermal conductivity, and dynamic viscosity were 0.04%, 0.01%, 0.16%, and 0.71%, respectively. The goodness-of-fit parameters [40] to assess the prediction quality of the correlations, such as *R*-square ($R^{2}$), adjusted *R*-square ($R_{\mathrm{adj}}^{2}$), sum of squares due to error (SSE), and the root mean squared error (RMSE), are listed in Table 6. Values of $R^{2}$ and $R_{\mathrm{adj}}^{2}$ closer to unity indicate that the fit is accurate, whereas for SSE and RMSE, a value closer to zero implies a better fit.

## 4. Results and Discussion

**Figure 4:**
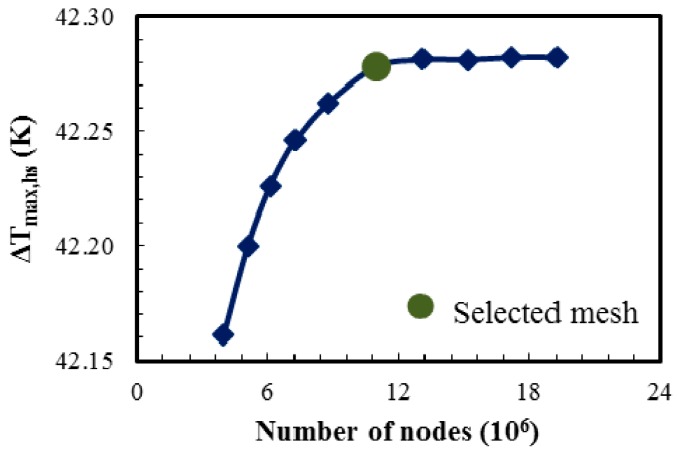
Grid independency test (PF design with single hotspot at the center at a total flow rate of 0.006 kg/s).

To create finite volumes in the computational domains for numerical calculations, unstructured tetrahedral and hexahedral elements were generated in the solid and fluid computational domains, respectively (with fine meshes near the walls of the fluid domain to resolve the boundary layer flow). A comprehensive grid sensitivity analysis was performed for consistent grid-independent results. Double-layer PF configuration with a single hotspot was selected for grid analysis at total flow rate of 0.006 kg/s. A wide range of grid nodes were tested; the number of nodes was varied from 3.9 million to 19.2 million. A grid with about 11 million nodes was determined to be optimum grid as no noticeable change in the results was observed with further increase in the grid density as shown in Figure 4 (13.1 million nodes showed 0.007% and 0.154% changes in the maximum temperature rise and the pressure drop, respectively, as compared to 11 million nodes and a grid with 8.7 million nodes showed 0.04% and 0.4% changes in the maximum temperature rise and the pressure drop, respectively, as compared to 11 million nodes).

**Figure 5:**
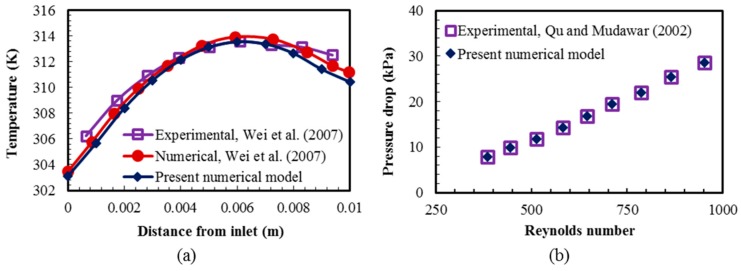
Validations of numerical results compared with: (**a**) experimental and numerical results obtained by Wei et al. [17] in a double-layer counter-flow heat sink for average temperature variation at the bottom surface in the flow direction; and (**b**) experimental results obtained by Qu and Mudawar [46] for the pressure drop in a rectangular microchannel with a heat flux of 200 W/cm^2^.

The numerical results using temperature-dependent fluid properties were validated with the previous numerical and experimental results [17,46] for the temperature distribution and the pressure drop, as shown in Figure 5. The present numerical results show good agreements with the numerical and experimental results presented by Wei et al. [17] for the temperature variation at the base of a double-layer counter-flow heat sink with rectangular microchannels as shown in Figure 5a. Qu and Mudawar [46] presented experimental results for the pressure drop within a rectangular microchannel. Figure 5b shows good agreements between the present numerical and previous experimental data for the pressure drop in a range of Reynolds number.

In the present study, PF, CF, and TF heat sink designs were selected for the performance analysis under three randomly generated non-uniform heating schemes. The cross-channel heat sink design with the TF configuration considered in the present work showed the lowest thermal resistance as compared to various other cross-channel and parallel-channel designs in the previous work [24]. The maximum increase in temperature at the hotspots, the pressure drop variation, the thermal resistance and the temperature variation among all the hotspots were selected as the performance parameters to be evaluated. A total flow rate range of 0.006 to 0.014 kg/s (Re range from 400 to 933) was used to analyze the performance variation with flow rate. The minimum value of flow rate was selected so that the maximum temperature rise at the hotspots became below the recommended temperature range for reliable performance of a microprocessor [7].

**Figure 6:**
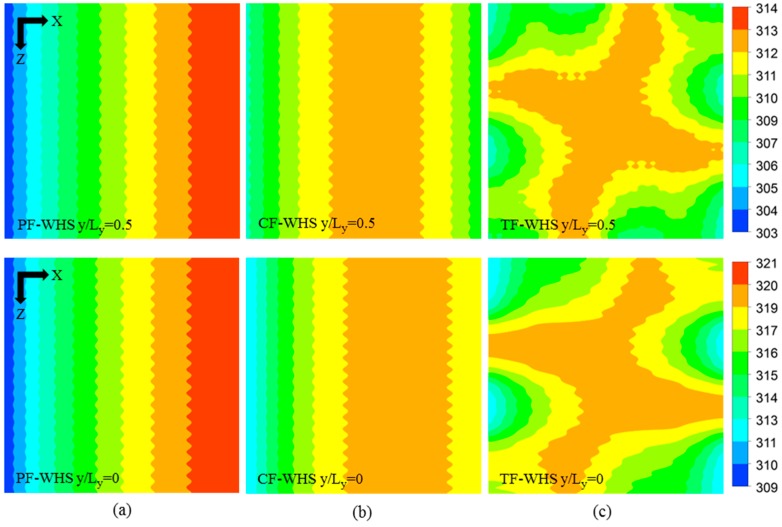
Temperature contours at the base (*y*/*L_y_* = 0) and at the middle height (*y*/*L_y_* = 0.5) of the heat sinks without hotspots at total flow rate of 0.01 kg/s: (**a**) PF-WHS; (**b**) CF-WHS; and (**c**) TF-WHS.

The local temperature contours at *y*/*L_y_* = 0 (bottom) and *y*/*L_y_* = 0.5 (middle) for the three heat sinks without hotspots (PF-WHS, CF-WHS, and TF-WHS) at total flow rate of 0.01 kg/s are shown in Figure 6. In the case of PF heat sink, the hottest zone is observed near the outlet region for both the bottom (*y*/*L_y_* = 0) and the top layers (*y*/*L_y_* = 0.5) (Figure 6a). Due to the opposite flow arrangement in the top layer of the CF heat sink, the hottest zone is almost at the center in the top layer, however, in the bottom layer, the hottest zone is still closer to the outlet with a slight shift towards the center (Figure 6b). The hottest zone in the TF heat sink is concentrated around the central region for both the bottom and the top layer as shown in Figure 6c. The TF heat sink showed lower thermal resistance and better temperature uniformity as compared to the PF and CF heat sinks in the previous work [24]. The better performance of the TF heat sink was attributed to the ubiquitous heat transfer present all over the heat sink as a result of quarterly opposite flow in the same layer and the transversal flow arrangement between the bottom and the top layers. The detailed analysis was presented by Ansari and Kim [24]. The wavy pattern observed in the temperature contours was due to the difference in conduction heat transfer in the heat sink walls and convection heat transfer in the channels. The total thermal resistance and maximum temperature increase at the bottom surface of the heat sinks without hotspots are listed in Table 7. The TF heat sink showed a 7.5% and 0.1% lower thermal resistance (and also temperature increase at the bottom surface) as compared to the PF and CF heat sinks, respectively. A variation in total pressure drop was observed among the PF, CF, and TF heat sinks even though all the design parameters were exactly the same [24]. This variation in the pressure drop was due to the consideration of variable fluid properties. The CF heat sink showed the lowest total pressure drop among the three heat sinks due to lowest average fluid temperature in both the layers [24].

**Figure 7:**
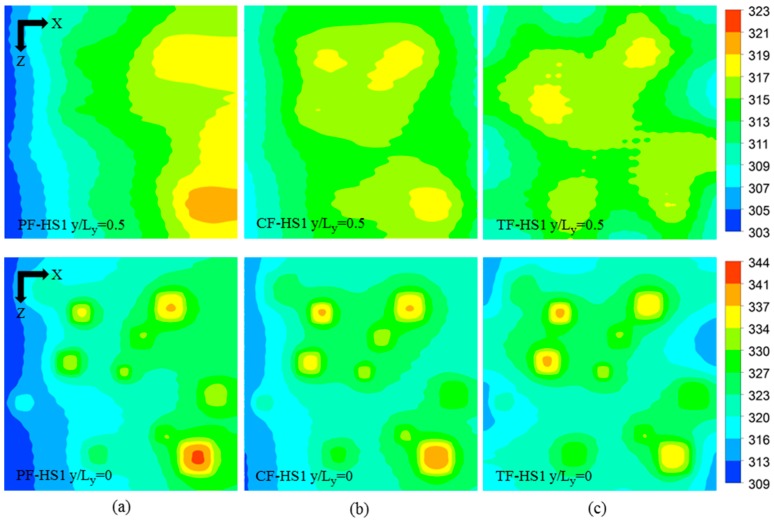
Temperature contours at the base (*y*/*L_y_* = 0) and at the middle height (*y*/*L_y_* = 0.5) of the heat sinks with the hotspot scheme HS1 at the total flow rate of 0.01 kg/s: (**a**) PF-HS1; (**b**) CF-HS1; and (**c**) TF-HS1.

**Figure 8:**
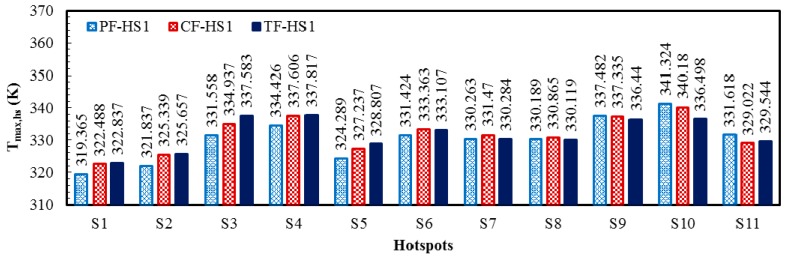
Maximum temperatures at the hotspots of PF, CF, and TF heat sinks with the hotspot scheme HS1 at the total flow rate of 0.01 kg/s.

The local temperature contours for the three heat sink designs (PF, CF, and TF) of the HS1 scheme (PF-HS1, CF-HS1, and TF-HS1), at total flow rate of 0.01 kg/s, are shown in Figure 7. The temperature contours are plotted at the bottom (*y*/*L_y_* = 0) and middle height of the heat sink (*y*/*L_y_* = 0.5). The comparison among the maximum temperatures at the hotspots of the PF, CF, and TF with the HS1 scheme is shown in Figure 8. The PF heat sink has a large temperature gradient in the direction of flow (Figure 6a), which causes overcooling of the hotspots (S_1_, S_2_, …) close to the inlet (*x* = 0) and undercooling of the hotspots (S_11_, S_10_, …) near the outlet (*x* = *L_x_*) creating large temperature difference between hotspots (Figure 7a). Due to the opposite flow arrangement in the top layer, the CF heat sink shows lower temperatures at the hotspots, S_10_ and S_11_, as compared to the PF heat sink (Figure 8). The TF heat sink shows relatively uniform temperature distribution all over the heat sink (Figure 6c). Due to the uniform temperature distribution in the TF heat sink, the hotspots near *x* = 0 are not overcooled (exhibits higher temperatures than the PF and CF heat sinks) and the hotspots near *x* = *L_x_* are not undercooled (exhibits lower temperatures than the PF and CF heat sinks) (Figure 8). The maximum temperatures in the PF and CF heat sinks are observed at the hotspot S10, whereas the maximum temperature in the TF heat sink is found at the hotspot S4, as shown in Figure 7 and Figure 8. Table 8 shows the maximum temperature rise at the hotspots, temperature variation between the hotspots, and the total thermal resistance for the HS1 scheme. The PF heat sink exhibits the highest temperature variation of 21.959 K among the hotspots. The CF heat sink exhibits a temperature variation of 17.692 K, which is 24.1% lower than that of the PF heat sink. Due to ubiquitous temperature distribution, the TF heat sink shows the lowest temperature variation among the three heat sinks (14.980 K), which is 46.6% and 18.1% lower than those of the PF and CF heat sinks, respectively. Along with significantly lower temperature variation among hotspots, the TF heat sink exhibits 9.3% and 6.2% lower thermal resistance (or maximum temperature rise) as compared to those of the PF and CF heat sinks, respectively.

**Figure 9:**
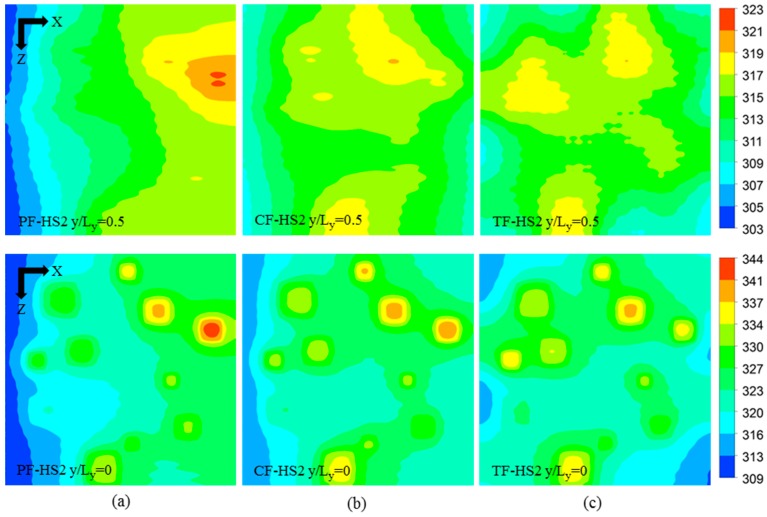
Temperature contours at the base (*y*/*L_y_* = 0) and at the middle height (*y*/*L_y_* = 0.5) of the heat sinks with the hotspot scheme HS2 at the total flow rate of 0.01 kg/s: (**a**) PF-HS2; (**b**) CF-HS2; and (**c**) TF-HS2.

**Figure 10:**
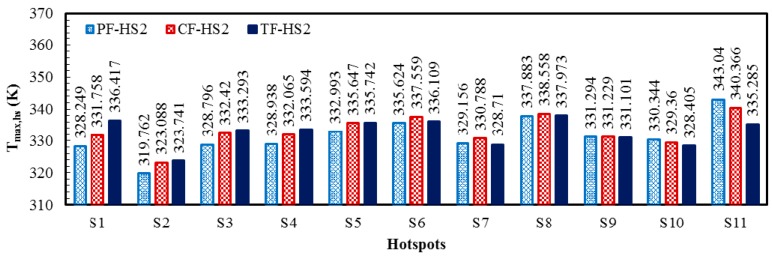
Maximum temperatures at the hotspots of PF, CF, and TF heat sinks with the hotspot scheme HS2 at the total flow rate of 0.01 kg/s.

Figure 9 shows the local temperature contours for the three heat sink designs (PF, CF, and TF) of the HS2 scheme (PF-HS2, CF-HS2, and TF-HS2), at total flow rate of 0.01 kg/s. The temperature contours are plotted at the bottom (*y*/*L_y_* = 0) and middle height of the heat sink (*y*/*L_y_* = 0.5). The maximum temperature rise at the hotspots, the temperature variation between the hotspots, and the total thermal resistance for the HS2 scheme are shown in Table 9. All the heat sinks with the HS2 scheme show the results similar to those for the HS1 scheme. The PF heat sink exhibits lower temperatures at the hotspots closer to the inlet due to overcooling, and due to undercooling the hotspots closer to outlet exhibits higher temperatures, producing 23.278 K temperature variation among the hotspots (Table 9). The temperature variation among hotspots in the CF heat sink is 17.278 K, which is 34.7% lower than that of the PF heat sink (Table 9). The TF heat sink exhibits the lowest temperature variation of 14.232 K, which is 63.6% and 21.4% lower than those of the PF and CF heat sinks, respectively (Table 9). Thermal resistance (or maximum temperature rise) is also lower for the TF heat sink, which is 13.3% and 6.3% lower than those of the PF and CF heat sinks, respectively (Table 9). The maximum temperatures in the PF and CF heat sinks occur at the hotspot S11 whereas the maximum temperature in the TF heat sink is found at the hotspot S8, as shown in Figure 9 and Figure 10.

**Figure 11:**
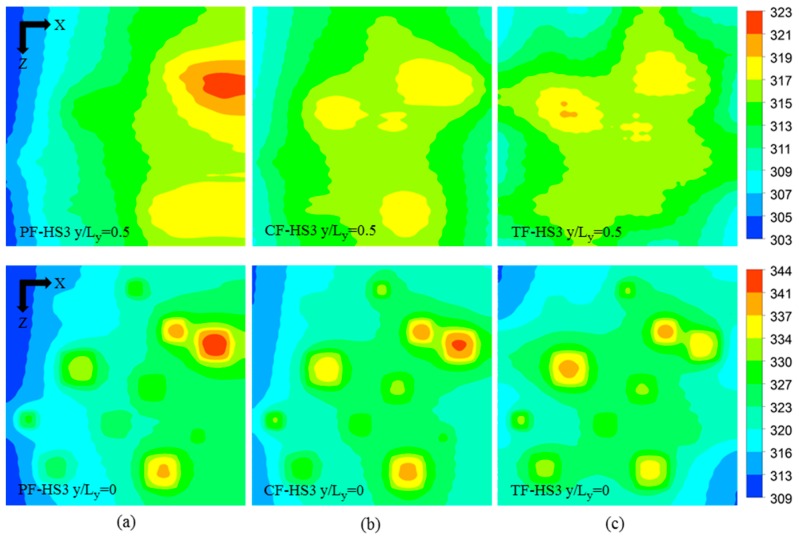
Temperature contours at the base (*y*/*L_y_* = 0) and at the middle height (*y*/*L_y_* = 0.5) of the heat sinks with the hotspot scheme HS3 at the total flow rate of 0.01 kg/s: (**a**) PF-HS3; (**b**) CF-HS3; and (**c**) TF-HS3.

**Figure 12:**
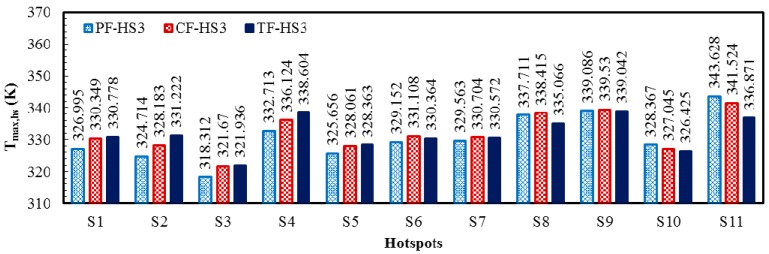
Maximum temperatures at the hotspots of PF, CF, and TF heat sinks with the hotspot scheme HS3 at the total flow rate of 0.01 kg/s.

The local temperature contours for all the heat sink designs (PF, CF, and TF) of the HS3 scheme (PF-HS3, CF-HS3, and TF-HS3), at total flow rate of 0.01 kg/s are shown in Figure 11. The temperature contours are plotted at the bottom (*y*/*L_y_* = 0) and middle height of the heat sink (*y*/*L_y_* = 0.5). The results are consistent with those for the HS1 and HS2 schemes. The TF heat sink exhibits the lowest temperature variation of 17.106 K, which is 48.0% and 16.1% lower than those of the PF (25.316 K) and CF (19.854 K) heat sinks, respectively (Table 10). The thermal resistance (or maximum temperature rise) of the TF heat sink is 11.7% and 6.4% lower than those of the PF and CF heat sinks, respectively (Table 10). The maximum temperatures at the hotspots are shown in Figure 12, which exhibits the highest temperatures at the hotspot S11 for the PF and CF heat sinks and at the hotspot S9 for the TF heat sink.

**Figure 13:**
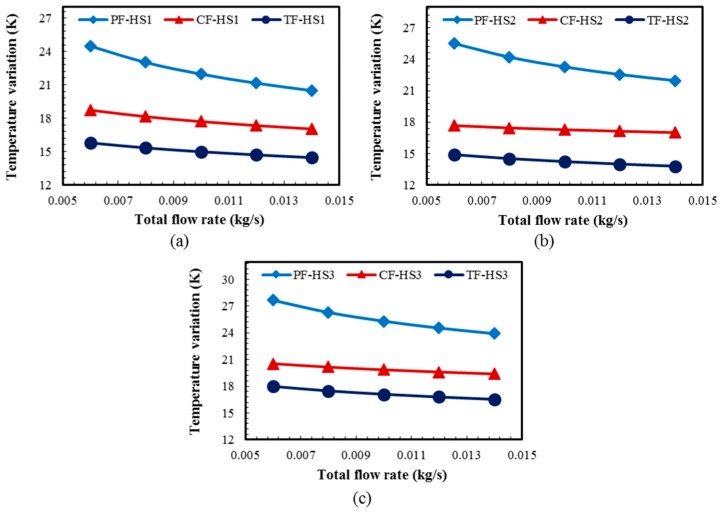
Temperature variation among the hotspots for different total flow rates for the PF, CF, and TF heat sinks (total flow rate varied from 0.006 to 0.014 kg/s): (**a**) HS1; (**b**) HS2; and (**c**) HS3.

Figure 13 shows the temperature variations among the hotspots for different total flow rate (in a range from 0.006 to 0.014 kg/s) for all the heat sink designs with the HS1, HS2, and HS3 schemes. The TF heat sink exhibits the lowest temperature variations for all the three hotspot schemes (HS1, HS2, and HS3) throughout the flow rate range, while the PF heat sink exhibits the highest temperature variations (Figure 13). The PF heat sink exhibits the highest decrease in the temperature variation with the increase in the total flow rate, whereas the CF and TF heat sinks show similar trends (Figure 13).

**Figure 14:**
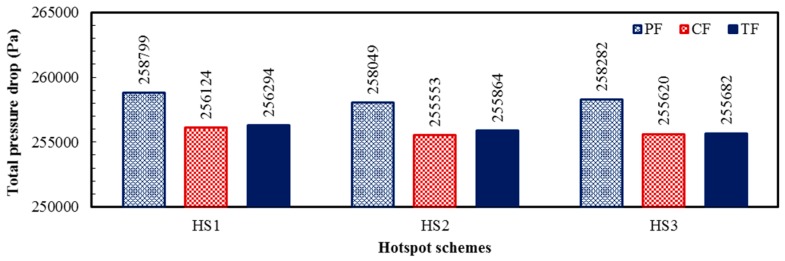
Total pressure drops in the PF, CF, and TF heat sink designs for the HS1, HS2, and HS3 hotspot schemes at the total flow rate of 0.01 kg/s.

Although all the heat sinks (PF, CF, and TF) were designed using exactly same microchannels, a slight variation in the pressure drop is observed in Table 11. The variation in the pressure drop was caused by the temperature-dependent fluid properties. With the increase in fluid temperature, the pressure drop reduces [47,48]. Table 11 shows the pressure drop and average fluid temperature variation in each layer of heat sink at the total flow rate of 0.01 kg/s. The pressure drops in the bottom layer (Δ*p*_bl_) are lower than that of the top layer (Δ*p*_tl_) in all the cases, because the average fluid temperatures in the bottom layer are higher than those of the top layer (Table 11). The total pressure drops in all the heat sinks (PF, CF, and TF)—with all the hotspot schemes (HS1, HS2, and HS3) at the total flow rate of 0.01 kg/s are shown in Figure 14. The lowest total pressure drop is exhibited by the CF heat sink for all the hotspots schemes, because of the highest average fluid temperature in each layer as compared to those of the PF and TF heat sinks (Figure 14 and Table 11). On the other hand, the PF heat sink exhibits the lowest fluid temperature in each layer resulting in the highest total pressure drop. The relative pressure drop variation among the three heat sink designs is only about 1% for all the hotspot schemes at the total flow rate of 0.01 kg/s. The total pumping power is minimum for the CF-HS2, and maximum for the PF-HS1, but the total variation is less than 1 mW as shown in Table 11.

## 5. Conclusions

In this study, non-uniform heating with random hotspots was introduced for the analysis of double-layer microchannel heat sinks. Eleven hotspots were randomly generated using LHS to constitute three sets of hotspot schemes for three double-layer heat sinks: two heat sinks from the double-layer parallel-channel design (PF and CF) and one heat sink from the cross-channel design (TF). The entire heat sink was comprised of two layers with 20 microchannels in each layer. All the microchannels had same dimensions. The heat flux, area, and location (*x* and *z*) of the hotspots were selected as the design parameters. Three-dimensional conjugate heat transfer analysis was performed using 3-D Navier–Stokes equations. The thermophysical properties of water (i.e., the density, specific heat, thermal conductivity, and dynamic viscosity) were allowed to change with temperature. Polynomial correlations between the thermophysical properties of water and temperature in the property table were developed and verified in this work. The entire heat sink was selected as computational domain. The total flow rate for analysis was varied in a range from 0.006 to 0.014 kg/s (*Re* range from 400 to 933). The numerical model was validated using experimental data for pressure drop (for rectangular channel single-layer heat sink) and average temperature variation at the base (for rectangular channel double-layer counter-flow heat sink). Among the heat sinks without hotspots, the lowest thermal resistance was exhibited by the TF heat sink. The TF heat sink design also exhibited the lowest thermal resistance, maximum temperature rise among the hotspots, and minimum temperature variation among the hotspots for all the three hotspot heating schemes (HS1, HS2, and HS3) at all the flow rates. The PF heat sink exhibited the largest change in the temperature variation with the change of the flow rate as compared to the CF and TF heat sinks for all the three heating schemes. The lowest pressure drop was exhibited by the CF heat sink (with and without hotspots) due to the highest average fluid temperature in both the layers. The maximum relative variation in pressure drop among the heat sinks under three heating schemes was only about 1% at total flow rate of 0.01 kg/s. From the overall estimation for all the three random heating schemes, it can be concluded that the TF heat sink can be the best choice for cooling of a microchip with multiple hotspots as it exhibits the lowest thermal resistance (also the lowest maximum temperature rise at the hotspots) and the lowest temperature variation among the hotspots. The random hotspot analysis presented in this work is expected to be useful to assess the performance of heat sinks with realistic heating conditions of advance multicore processors.

## Figures and Tables

**Table 1:** Microchannel dimensions (mm).

*W*_c_	*H*_c_	*W*_w_	*W*_b1_	*W*_b2_	*W*_cp_	*L_x_*	*L_y_*	*L_z_*
0.25	0.50	0.25	0.20	0.20	0.20	10	1.6	10

**Table 2:** Hotspot parameters and their ranges.

	*x* (mm)	*z* (mm)	*A*_hs_ (mm^2^)	*q*_hs_ (W/cm^2^)
LB	0.75	0.75	0.25	200
UB	9.25	9.25	2.25	600

**Table 3:** Hotspots for the HS1 scheme.

Hotspot	*x* (mm)	*z* (mm)	*A*_hs_ (mm^2^)	*q*_hs_ (W/cm^2^)
S1	0.80	6.22	0.41	374
S2	1.56	1.36	2.06	225
S3	2.74	4.51	1.03	466
S4	3.28	2.38	0.73	558
S5	3.90	8.42	1.26	256
S6	5.12	4.92	0.30	582
S7	5.84	3.44	0.80	311
S8	6.80	7.54	0.49	359
S9	7.06	2.08	1.44	403
S10	8.18	8.56	1.85	435
S11	8.94	5.86	1.54	258

**Table 4:** Hotspots for the HS2 scheme.

Hotspot	*x* (mm)	*z* (mm)	*A*_hs_ (mm^2^)	*q*_hs_ (W/cm^2^)
S1	1.34	4.62	0.63	526
S2	1.58	6.78	1.53	211
S3	2.30	2.00	2.18	325
S4	3.22	4.22	1.35	321
S5	4.12	9.16	1.90	343
S6	5.24	0.80	0.51	576
S7	5.46	8.16	0.39	386
S8	6.50	2.50	1.25	437
S9	7.10	5.48	0.34	495
S10	7.78	7.46	1.08	289
S11	8.82	3.32	0.93	547

**Table 5:** Hotspots for the HS3 scheme.

Hotspot	*x* (mm)	*z* (mm)	*A*_hs_ (mm^2^)	*q*_hs_ (W/cm^2^)
S1	1.34	4.62	0.63	594
S2	1.58	6.78	1.53	300
S3	2.30	2.00	2.18	201
S4	3.22	4.22	1.35	379
S5	4.12	9.16	1.90	237
S6	5.24	0.80	0.51	488
S7	5.46	8.16	0.39	272
S8	6.50	2.50	1.25	393
S9	7.10	5.48	0.34	503
S10	7.78	7.46	1.08	312
S11	8.82	3.32	0.93	452

**Table 6:** Goodness-of-fit parameters for the temperature-dependent property correlations.

Equations	$R^{2}$	$R_{adj}^{2}$	SSE	RMSE
ρ_w_	0.9997	0.9997	1.1200	0.2567
*C*_p,w_	0.9995	0.9993	1.9340	0.3477
*k*_w_	0.9999	0.9998	3.732 × 10^−6^	4.686 × 10^−4^
µ_w_	1.0000	1.0000	9.979 × 10^−11^	2.497 × 10^−6^

**Table 7:** Total thermal resistances and maximum temperature increases at the base of the PF, CF, and TF heat sinks without hotspot (WHS) at the total flow rate of 0.01 kg/s.

Temperature (K)	Heat Sink Designs		
	PF-WHS	CF-WHS	TF-WHS
Δ*T*_max,b_ (K)	20.827	19.390	19.374
			7.5% ↓ _PF-WHS_
			0.1% ↓ _CF-WHS_
*R*_th,tot_ (K/W)	0.2083	0.1939	0.1937
			7.5% ↓ _PF-WHS_
			0.1% ↓ _CF-WHS_

**Table 8:** Total thermal resistances and maximum temperature increases at the hotspots of the PF, CF, and TF heat sinks for the hotspot scheme HS1 at the total flow rate of 0.01 kg/s.

Temperature (K)	Heat Sink Designs		
	PF-HS1	CF-HS1	TF-HS1
Δ*T*_max,S1_	19.365	22.488	22.837
Δ*T*_max,S2_	21.837	25.339	25.657
Δ*T*_max,S3_	31.558	34.937	37.583
Δ*T*_max,S4_	34.426	37.606	37.817
Δ*T*_max,S5_	24.289	27.237	28.807
Δ*T*_max,S6_	31.424	33.363	33.107
Δ*T*_max,S7_	30.263	31.470	30.284
Δ*T*_max,S8_	30.189	30.865	30.119
Δ*T*_max,S9_	37.482	37.335	36.440
Δ*T*_max,S10_	41.324	40.180	36.498
Δ*T*_max,S11_	31.618	29.022	29.544
Δ*T*_min,spot_	19.365	22.488	22.837
Δ*T*_max,spot_	41.324	40.180	37.817
			9.3% ↓ _PF-HS1_
			6.2% ↓ _CF-HS1_
Variation (range) Δ*T*_max,spot_−*Δ*T_min,spot_	21.959	17.692	14.980
			46.6% ↓ _PF-HS1_
			18.1% ↓ _CF-HS1_
*R*_th,tot_ (K/W)	0.3175	0.3087	0.2906
			9.3% ↓ _PF-HS1_
			6.2% ↓ _CF-HS1_

**Table 9:** Total thermal resistances and maximum temperature increases at the hotspots of the PF, CF, and TF heat sinks for the hotspot scheme HS2 at the total flow rate of 0.01 kg/s.

Temperature (K)	Heat Sink Designs		
	PF-HS2	CF-HS2	TF-HS2
Δ*T*_max,S1_	28.249	31.758	36.417
Δ*T*_max,S2_	19.762	23.088	23.741
Δ*T*_max,S3_	28.796	32.420	33.293
Δ*T*_max,S4_	28.938	32.065	33.594
Δ*T*_max,S5_	32.993	35.647	35.742
Δ*T*_max,S6_	35.624	37.559	36.109
Δ*T*_max,S7_	29.156	30.788	28.710
Δ*T*_max,S8_	37.883	38.558	37.973
Δ*T*_max,S9_	31.294	31.229	31.101
Δ*T*_max,S10_	30.344	29.360	28.405
Δ*T*_max,S11_	43.040	40.366	35.285
Δ*T*_min,spot_	19.762	23.088	23.741
Δ*T*_max,spot_	43.040	40.366	37.973
			13.3% ↓ _PF-HS2_
			6.3% ↓ _CF-HS2_
Variation (range) Δ*T*_max,spot_–Δ*T*_min,spot_	23.278	17.278	14.232
			63.6% ↓ _PF-HS2_
			21.4% ↓ _CF-HS2_
*R*_th,tot_ (K/W)	0.3255	0.3053	0.2872
			13.3% ↓ _PF-HS2_
			6.3% ↓ _CF-HS2_

**Table 10:** Total thermal resistances and maximum temperature increases at the hotspots of the PF, CF, and TF heat sinks for the hotspot scheme HS3 at the total flow rate of 0.01 kg/s.

Temperature (K)	Heat Sink Designs		
	PF-HS3	CF-HS3	TF-HS3
Δ*T*_max,S1_	26.995	30.349	30.778
Δ*T*_max,S2_	24.714	28.183	31.222
Δ*T*_max,S3_	18.312	21.670	21.936
Δ*T*_max,S4_	32.713	36.124	38.604
Δ*T*_max,S5_	25.656	28.061	28.363
Δ*T*_max,S6_	29.152	31.108	30.364
Δ*T*_max,S7_	29.563	30.704	30.572
Δ*T*_max,S8_	37.711	38.415	35.066
Δ*T*_max,S9_	39.086	39.530	39.042
Δ*T*_max,S10_	28.367	27.045	26.425
Δ*T*_max,S11_	43.628	41.524	36.871
Δ*T*_min,spot_	18.312	21.670	21.936
Δ*T*_max,spot_	43.628	41.524	39.042
			11.7% ↓ _PF-HS3_
			6.4% ↓ _CF-HS3_
Variation (range) Δ*T*_max,spot_–Δ*T*_min,spot_	25.316	19.854	17.106
			48.0% ↓ _PF-HS3_
			16.1% ↓ _CF-HS3_
*R*_th,tot_ (K/W)	0.3023	0.2877	0.2705
			11.7% ↓ _PF-HS3_
			6.4% ↓ _CF-HS3_

**Table 11:** Average fluid temperatures (in each layer) and pressure drops (in each layer and total) of the PF, CF, and TF heat sinks with the hotspot schemes HS1, HS2, and HS3 at the total flow rate of 0.01 kg/s.

Design	*T*_avg,f,bl_ (K)	*T*_avg,f,tl_ (K)	Δ*p*_bl_ (Pa)	Δ*p*_tl_ (Pa)	Δ*p*_tot_ = Δ*p*_bl_ + Δ*p*_tl_ (Pa)	*P*_tot_ (W) × 10^−3^
PF-HS1	308.623	305.973	126,707	132,092	258,799	64.894
CF-HS1	309.520	307.014	125,429	130,695	256,124	64.224
TF-HS1	309.514	306.802	125,477	130,816	256,293	64.226
PF-HS2	308.879	306.166	126,302	131,747	258,049	64.706
CF-HS2	309.748	307.123	125,105	130,448	255,553	64.080
TF-HS2	309.635	306.905	125,243	130,621	255,864	64.158
PF-HS3	308.789	306.090	126,422	131,861	258,283	64.765
CF-HS3	309.693	307.127	125,151	130,469	255,620	64.097
TF-HS3	309.705	306.998	125,132	130,550	255,682	64.113

## Nomenclature

*A*_b_	total area of the heat sink base (m^2^)
*A*_bg_	area of background base (excluding hotspot area) (m^2^)
*A*_hs_	area of single hotspot (m^2^)
*A*_c_	microchannel cross-sectional area (m^2^)
BL	bottom layer
CF	counter flow
*C*_p_	specific heat (J/kg·K)
*D*_h_	hydraulic diameter of the channel (m)
*h*_conv_	convective heat transfer coefficient (W/m^2^·K)
*H*_c_	microchannel height (m)
HS1, HS2, HS3	three random hotspot layout schemes
*k*	thermal conductivity (W/m·K)
LB	lower bound
*L*_s_	length of hotspot (m)
*L_x_*, *L_y_*, *L_z_*	length, total height, and total width of the heat sink, respectively (m)
$m_{\mathrm{tot}}$	total mass flow rate (kg/s)
$n_{\mathrm{ch}}$	total numbers of channels
*Δp*_ch_	pressure drop in one channel (Pa)
*P*_tot_	total pumping power required for a heat sink (W)
PF	parallel flow
$q_{\mathrm{hs}},q_{b}$, $q_{\mathrm{bg}}$	heat fluxes applied at the hotspot, base, and background base, respectively (W/m^2^)
*Q*_tot_	total heat applied at the base (W)
*Re*	Reynolds number
*R*_th,tot_	total thermal resistance (K/W)
*T*	temperature (K)
TL	top layer
TF	transverse flow
*u*_avg_	average fluid velocity (m/s^2^)
UB	upper bound
$\bar{V}$	velocity vector
*W*_b1_	thickness of base at the bottom (m)
*W*_b2_	thickness of base between the bottom and top layer (m)
*W*_c_	microchannel width (m)
*W*_cp_	thickness of the cover plate (m)
W_s_	width of a hotspot (m)
*W*_w_	width of microchannel walls (m)
*X*, *Y*, *Z*	orthogonal coordinate system

## Subscripts

avg	average value
b	base of heat sink
bl	bottom layer
f	fluid
HS	hotspot
in	inlet
max	maximum value
s	solid
tl	top layer
tot	total
w	water
WHS	without hotspot

## Greek Symbols

µ	dynamic viscosity of fluid (kg/m·s)
ρ	density of fluid (kg/m^3^)
